# Supplementary material for: Evaluating Transmission Heterogeneity and Super-Spreading Event of COVID-19 in a Metropolis of China
Source: Int J Environ Res Public Health. 2020 May 24;17(10):3705. doi: 10.3390/ijerph17103705 (PMC7277812; doi:10.3390/ijerph17103705)
Supplement: Supplementary file 1 [file ijerph-17-03705-s001.zip › Supplementary/Supplementary Material.pdf]

Supplementary materials for "Evaluating transmission heterogeneity and super-spreading event of COVID-19 in a metropolis of China"

Yunjun Zhang [1,†], Yuying Li [1,†], Lu Wang [2], Mingyuan Li [3] and Xiaohua Zhou [1,2,4]*
[1] *Department of Biostatistics, School of Public Health, Peking University, Xueyuan Road, 100191, Beijing, China*
[2] Beijing International Center for Mathematical Research, Peking University, Yiheyuan Road, 100871, Beijing, China
[3] *School of Mathematical Sciences, Peking University, Peking University, Yiheyuan Road, 100871, Beijing, China*
[4] *Center for Statistical Science, Peking University, Yiheyuan Road, 100871, Beijing, China*
Correspondence: azhou@math.pku.edu.cn
†These authors contributed equally to this work.

The supplementary materials are organized as follows. Section A describes detail information of three type of transmission chain. Section B gives the detail information for the construction of statistical model. Section C presents the EM algorithm for the estimation of parameters R_0 and k. Section D gives technical details for the construction of bootstrap confidence interval (CI) of the estimates from EM algorithm.

A. Description of different transmission chain

The details of the transmission chains grouped based on individual-case information in-Tianjin are given below.

a. Simple transmission chain

There are 36 size-one chains, 5 size-two chains and 2 size-four chains. The detailed information about two size-four chain as following:

- *Chain A* The case with primary ID 32, a 28-year-old man, confirmed on January 31st. He infected three relatives, confirmed durg February 1st to February 3rd. No more cases have been detected with epidemiological links with these four individuals.

- *Chain B* The case with primary ID 82, a 47-year-old man, confirmed on February 8th. He infected two relatives and one colleague, confirmed on February 9th, 11th and 21st respectively.

b. Ordinary transmission chain

- *EMU depot transmission chain* This was the first ordinary transmission chain reported in Tianjin, including 16 cases. The primary case with primary ID 2 was a 57-year-old man who works in Tianjin EMU depot, and has a history of business trip to Wuhan. He confirmed on January 21st and directly infected six colleagues in close contact with

him and indirectly infected 9 cases. His six colleagues successively confirmed between January 23rd and February 3rd. These six cases, as secondary cases in this transmission chain, successively infected other colleagues or their relatives. A third-generation case in this chain died, and he has a history of hypertension for 30 years, cerebral infarction for 10 years, and diabetes for 18 years.

- *Department store chain* This was the largest transmission chain reported in Tianjin, including 45 cases. Among of cases, 5 cases are salesclerksand 40 cases are shoppers or their relatives. The primary case is a 43-year-old woman, working in the department store. She had no history of travel or residence in Wuhan before the onset of illness. However, she travelled to Beijing from January 16th to 17th, and worked closely with a fever customer in a jewelry mall in Beijing. Sine the specific transmission routes cannot be determined in department store, except the primary case, we cannot distinguish between different generations of other cases. Thus, in Figure 2, we used a dotted box to indicate that all cases in this chain have the potential to infect others.
- *Chain C* The case with primary ID 3, a 68-year-old woman, confirmed on January 21th. She worked at Wuhan city and showed symptoms on January 14th. She infected case 9 which showed symptoms on January 24th and confirmed on January 25th. Then case 9 infected two relatives of him.
- *Chain D* The case with primary ID 93, a 50-year-old woman, confirmed on February 7th. She infected 3 relatives of her. Three neighbors were infected when visited her at her home and then transmit virus to their family members.
- *Chain E* The case with primary ID 102, a 49-year-old man, confirmed on January 11th. He infected his wife who confirmed on January 12th and his wife infected her sister who confirmed on January 14th.

c. Complex transmission chain

- *Chain F* A couple (aged 49 and 50 respectively) with primary ID 95 and 101 infected three of their relatives. The couple stayed closed all the time, and had equal chance of infecting their relatives. In addition, they both showed first symptoms on January 1st. So we regarded them both as primary cases. The couple were confirmed on February 10th and 11th. Their relatives were confirmed on February 11th, 13th and 16th.
- *Chain G* A couple (aged 80 and 85 respectively) with primary ID 100 and 113 infected three of their relatives. Both of them had a contact history with a person returning to Tianjin from Shanghai and we regarded them as primary cases. The couple confirmed on February 11th and 13th. The relatives of them confirmed on January 11th and 12th.

c. Complex transmission chain

- *Chain H* A couple with primary ID 95 and 101 infected three of their relatives. The couple stayed closed all the time, and had equal chance of infecting their relatives. In addition, they both showed symptoms on January 1. So we regarded them both as

primary cases. The couple confirmed on February 10 and 11. The relatives of them confirmed on February 11, 13 and 16.

- *Chain I* A couple with primary ID 100 and 113 infected three of their relatives. Both of them have a contact history with a person returning to Tianjin from Shanghai and we regarded them as primary cases. The couple confirmed on February 11 and 13. The relatives of them confirmed on January 11 and 12.

B. Statistical Model

Likelihood function

To quantify the transmission potential and degree of transmission heterogeneity for the COVID-19 outbreak in Tianjin, we adopt a likelihood-based approach proposed by [1] which characterizes both the mean and heterogeneity of individual infectiousness. In consideration of three different types of transmission chains included in the data set, we here use different methods to model each type of chains. Under the assumption that transmission chains are independent with each other, the overall likelihood function has the form

$$L\left(R,k\right) = L_I\left(R,k\right) L_{II}\left(R,k\right) L_{III}\left(R,k\right), \tag{S.1}$$

where R and k are parameters of interest, $L_I\left(R,k\right)$, $L_{II}\left(R,k\right)$, and $L_{III}\left(R,k\right)$ represent the likelihood function of the model for fitting simple, ordinary, and complex transmission chains, respectively. In the following, we present the ways to model each type of transmission chains and construct the corresponding likelihood functions.

Firstly, regarding the simple transmission chain, since the information of who-infected-whom has been recovered completely, we are able to directly model the offspring distribution. To be specific, let S denote the number of secondary cases caused by an infected individual, and assume that S follows a *Negative Binomial* distribution (referred to as the offspring distribution). Suppose there are s_i secondary cases directly caused by the i-th case included in simple transmission chains, for $i = 1, \cdots, n_I$, then the likelihood function of the model for fitting simple transmission chains can be written as

$$L_I\left(R,k\right) = \prod_{i=1}^{n_I} f_1(s_i; R, k), \tag{S.2}$$

where $f_1(s_i; R, k)$ is the probability mass function (pmf) of the *Negative Binomial* with mean R and dispersion parameter k, i.e.,

$$f_1(s_i; R, k) \stackrel{\text{def}}{=} Pr\left(S = s_i\right) = \frac{\Gamma\left(s_i + k\right)}{\Gamma\left(s_i + 1\right)\Gamma\left(k\right)} \left(\frac{k}{R+k}\right)^k \left(\frac{R}{R+k}\right)^{s_i},$$

in which Γ denotes the *Gamma* function.

We here consider the *Negative Binomial* to characterize the offspring distribution given its general formulation in that the negative binomial model also includes the conventional Poisson ($k \to \infty$) and geometric ($k = 1$) models as special cases. Consequently, the offspring distribution has the variance equals $R(1 + R/k)$, thus smaller values of k indicate greater

heterogeneity which means a preponderance of very small and very large chains (large chains are associated with super-spreading events), together with a low frequency of intermediate-size chains [2].

Secondly, for the ordinary transmission chain, it is inappropriate to model the offspring distribution due the uncertainty in transmission relationships between inner-generations and inter-generation, thus we consider the chain size distribution instead. We here approximate the transmission dynamics of infection through a Galton–Watson branching process [3]. Let Q denote the overall size of a transmission chain. According to [4, 1], the probability of a transmission chain with one primary case having an overall size of q equals

$$f_2(q;R,k) \stackrel{\text{def}}{=} Pr\left(Q=q\right) = \frac{\Gamma\left(kq+q-1\right)}{\Gamma\left(kq\right)\Gamma\left(q+1\right)}\left(\frac{R}{k}\right)^{(q-1)}\left(1+\frac{R}{k}\right)^{-kq-q+1}.$$

Assume that the j-th ordinary transmission chain has an overall size of q_j, for $j=1,\cdots,n_{II}$, then the likelihood function of the chain size model for ordinary transmission chains equals

$$L_{II}\left(R,k\right) = \prod_{j=1}^{n_{II}} f_2(q_j;R,k). \tag{S.3}$$

Thirdly, in handling a complex transmission chain with two primary cases, we regard it can be separated into two ordinary transmission chains, each of which is led by a primary case. The difficulty lies in that the exact size of each ordinary chain is unclear. We here consider the combinatorial method in [1] to deal with this ambiguity by allowing for all the possible combinations and treat the sum as an overall probability. Suppose the m-th complex transmission chain is with an overall size of r_m, and let m_1 and m_2 denote the sizes of two separated ordinary chains of the m-th complex chain, for $m=1,\cdots,n_{III}$. Then the probability of a complex transmission chain with size r_m is $\sum\limits_{m_1+m_2=r_m} f_2(m_1;R,k)f_2(m_2;R,k)$, and hence the likelihood of the model for complex transmission chains is

$$L_{III}\left(R,k\right) = \prod_{m=1}^{n_{III}} \left(\sum_{m_1+m_2=r_m} f_2(m_1;R,k)f_2(m_2;R,k)\right). \tag{S.4}$$

Notice that the complex transmission chain in Tianjin COVID-19 data contains at most two primary cases, thus we only need to consider all possible combinations of the chains with two primary cases. While this combinatorial method can be naturally extended to the more complicated chains with more than two primary cases.

Lastly, followed by equations (S.1), (S.2), (S.3), and (S.4), the overall likelihood function turns out to be

$$L\left(R,k\right) = \prod_{i=1}^{n_I} f_1(s_i;R,k)\cdot\prod_{j=1}^{n_{II}} f_2(q_j;R,k)\cdot\prod_{m=1}^{n_{III}}\left(\sum_{m_1+m_2=r_m} f_2(m_1;R,k)f_2(m_2;R,k)\right). \tag{S.5}$$

Up to now, we successfully build up a likelihood function to simultaneously accommodate the three different types of transmission chains, i.e, the simple, ordinary, and complex transmission chains. In this way, we are able to use the mixed-type data to get a comprehensive estimation of R and k at the same time.

Estimation and Inference of R and k

Next, we consider to estimate and make inference about R and k based on the derived likelihood function (S.5). It is straightforward to obtain the maximum likelihood estimates (MLE) of parameters R and k by maximizing the log-likelihood function. By taking the first derivative of $\log L(R,k)$ with respect to R and then equating it to zero to solve for R, one can easily get

$$\hat{R} = \frac{\sum_{i=1}^{n_I} s_i + \sum_{j=1}^{n_{II}} (q_j - 1) + \sum_{m=1}^{n_{III}} (r_m - 2)}{n_I + \sum_{j=1}^{n_{II}} q_j + \sum_{m=1}^{n_{III}} r_m} \stackrel{\text{def}}{=} \frac{N_o}{N}, \tag{S.6}$$

where N_o and N denote the total number of offspring infections and the total number of infections across all chains, respectively. The MLE of R is analytically tractable, while that of k is not thus it can only be derived by computational optimization. In our implementation, we apply the modified quasi-Newton method proposed by [5] to solve such optimization problem. In addition, we here adopt the likelihood profiling considered in [1] to determine the confidence intervals (CI) and confidence region for R and k.

C. EM algorithm for the estimation of R_0 and k

Besides the MLE method introduced in the main text, we also propose to adopt the EM algorithm for dealing with the complex transmission chain in the estimation of parameters R_0 and k. Given the size of transmission chain produced by each primary case is unknown, we here treat the size as a latent variable X. Since all chains are independent from each other, the latent variable for chains with the same size should share the same latent distribution. Hence, the latent variable X can be viewed as a function of chain size r_m, i.e., $X = X_{r_m} \in \{1, 2, \cdots, r_m - 1\}$. Then, the likelihood function for modeling the complex transmission chain with the latent variable X_{r_m} can be written as

$$L_{III,\text{EM}}(R_0, k; X_{r_m}) = \prod_{m=1}^{n_{III}} \left[f_2(X_{r_m}) f_2(r_m - X_{r_m}) \right], \tag{S.7}$$

and the overall likelihood turns out to be

$$L_{EM}(R_0, k; X_M) = \prod_{i=1}^{n_I} f_1(s_i) \prod_{j=1}^{n_{II}} f_2(r_j) \prod_{m=1}^{n_{III}} \left[f_2(X_{r_m}) f_2(r_m - X_{r_m}) \right]. \tag{S.8}$$

In practice, the EM algorithm are executed as follows.

Step 1. Start with initial values for parameters R_0 and k, denoted by R_0^0 and k^0, respectively. One possible way to achieve the initial value is fitting a negative binomial model for the offspring distribution to get the corresponding parameter estimate.

Step 2. Calculate the conditional distribution of $X = (X_3, X_4, \cdots)$,

$$Q(x) = \frac{L_{III,EM}(R_0^0, k^0; x)}{\sum_x L_{3,EM}(R_0^0, k^0; x)}, x_{r_m} \in \{1, 2, \cdots, r_m - 1\}$$

Step 3. Find the solution to the optimization problem

$$(R_0^*, k^*) = \text{argmax} \sum_x Q(x) \ln L_{EM}(R_0^0, k^0; x) \overset{\text{def}}{=} \text{argmax} J_Q(R_0^0, k^0)$$

Step 4. If the deduction of the target function in step 3 is greater than a small enough value ε, i.e.,

$$J_Q(R_0^0, k^0) - J_Q(R_0^*, k^*) \geq \varepsilon,$$

then we set $R_0^0 = R_0^*$, $k^0 = k^*$, and repeat step 2-4; otherwise, return (R_0^0, k^0) as the final solution.

C. Bootstrap Confidence Interval for the EM algorithm

To construct CI for the estimates of R_0 and k obtained from EM algorithm, we consider the bootstrap method. Assume the original data set contains N_1 simple transmission chains, N_2 ordinary transmission chains and N_3 complex transmission chains. Let $\hat{R}_0$ and $\hat{k}$ denote the estimator of R and k, respectively. We here generate bootstrap samples using stratified sampling method given there are three different types of transmission chains. The resampling procedure is conducted as follows.

Step 1. Set bootstrap size $B = 10000$.

Step 2. Calculate the probability mass function (pmf) of chain size for each ordinary and complex transmission chain, in which R_0 and k are substituted by their corresponding estimate. The pmf of the i-th ordinary and j-th complex transmission chain are denoted by f_2^i and f_3^j, respectively, for $i = 1, \ldots, N_2$ and $j = 1, \ldots, N_3$.

Step 3. Sum up $\{f_2^i\}_{i=1}^{N_2}$ and $\{f_3^j\}_{j=1}^{N_3}$ obtained from **Step 2** to get the cumulative distribution function denoted by F_2 and F_3, respectively.

Step 4. Generate N_1, N_2 and N_3 random samples from the negative binomial distribution, distribution F_2, and distribution F_3, respectively.

Step 5. Apply the EM algorithm to the bootstrap samples generated from **Step 4** to get estimates of R_0 and k.

Step 6. Repeat **Step 4-5** until achieve B bootstrap estimates. Denote $\hat{R}_0^r$ and $\hat{k}^r$ as the estimates from the r-th bootstrap samples, for $r = 1, \ldots, B$.

With the bootstrap estimates, we here construct corresponding bias-corrected CI for each parameter. To be specific, the lower and upper thresholds of R_0 are $\beta_{lower} = \Phi(2z + \Phi^{-1}(\alpha/2))$ and $\beta_{upper} = \Phi(2z + \Phi^{-1}(1 - \alpha/2))$, respectively, where Φ is the cdf of the standard normal distribution, Φ^{-1} is the inverse function of Φ, and $z = \Phi^{-1}(\frac{1}{B} \sum_{r=1}^{B} 1[R_0^{(r)} < \hat{R}_0])$ being the fraction of bootstrap estimates lower than $\hat{R}_0$. The lower and upper bounds of the bias-corrected interval are the β_{lower} and β_{upper} quantile of $\{\hat{R}_0^{(r)}\}_{r=1}^{B}$. The CI for k can be derived similarly.

In practice, the distribution of the estimator $\hat{k}$, approximated by the bootstrap, shows severe skewness (asymmetry). Therefore, we adopt the bias-correction method aiming to correct for the bias induced by this feature. The correction coefficient, z, measures how far the point estimate is from the median. When the sampling distribution is heavily skewed, the corrected lower and upper thresholds tends to deviate further from the traditional thresholds, i.e., $\alpha/2$ and $1-\alpha/2$.

Bootstrapping provides a way to derive CI for more complex estimators. Since the EM method requires maximizing likelihood with latent variables, likelihood ratios (LR) are difficult to calculate, and thus LR test does not apply. Bootstrap can overcome this complexity with slightly more computational costs.

From our results as shown in the main text, the bootstrap method gives narrower CIs than the LR. One possible reason is that the chi-square distribution, as the limit distribution of likelihood ratio, does not give good approximation to the sampling distribution of LR due to the data size being too small. Another possible factor is the sampling scheme, where we assumed constant number of chains of each type. This information have potential influence on the length of the interval.

It is worthwhile to point out that when original data size is too small, parametric bootstrap works better than nonparametric bootstrap, which resamples with replacement from original data itself, not from the estimated distribution. The latter will give biased results since the empirical distribution of the data is not close from the true one.

References

[1] Blumberg, Seth and Lloyd-Smith, James O. *Inference of R_0 and transmission heterogeneity from the size distribution of stuttering chains*, PLoS Computational Biology 2013, 9.

[2] Chowell, Gerardo, Abdirizak, Fatima and Lee, Sunmi et al.. *Transmission characteristics of MERS and SARS in the healthcare setting: a comparative study*, BMC medicine, 2015, 13

[3] Harris, Theodore Edward. *The theory of branching process*, 1964

[4] Nishiura, Hiroshi, Yan, Ping and Sleeman, Candace K et al.. *Estimating the transmission potential of supercritical processes based on the final size distribution of minor outbreaks*, Journal of theoretical biology, 2012, 294

[5] Byrd, Richard H, Lu, Peihuang and Nocedal, Jorge et al.. *A limited memory algorithm for bound constrained optimization*, SIAM Journal on scientific computing, 1995, 16
